# The future of genomics in polar and alpine cyanobacteria

**DOI:** 10.1093/femsec/fiy032

**Published:** 2018-02-23

**Authors:** Nathan A M Chrismas, Alexandre M Anesio, Patricia Sánchez-Baracaldo

**Affiliations:** 1 Bristol Glaciology Centre, School of Geographical Sciences, University of Bristol, University Road, Bristol, BS8 1SS, UK; 2 Marine Biological Association of the United Kingdom, The Laboratory, Citadel Hill, Plymouth, PL1 2PB, UK

**Keywords:** cyanobacteria, cryosphere, polar, alpine, genomics

## Abstract

In recent years, genomic analyses have arisen as an exciting way of investigating the functional capacity and environmental adaptations of numerous micro-organisms of global relevance, including cyanobacteria. In the extreme cold of Arctic, Antarctic and alpine environments, cyanobacteria are of fundamental ecological importance as primary producers and ecosystem engineers. While their role in biogeochemical cycles is well appreciated, little is known about the genomic makeup of polar and alpine cyanobacteria. In this article, we present ways that genomic techniques might be used to further our understanding of cyanobacteria in cold environments in terms of their evolution and ecology. Existing examples from other environments (e.g. marine/hot springs) are used to discuss how methods developed there might be used to investigate specific questions in the cryosphere. Phylogenomics, comparative genomics and population genomics are identified as methods for understanding the evolution and biogeography of polar and alpine cyanobacteria. Transcriptomics will allow us to investigate gene expression under extreme environmental conditions, and metagenomics can be used to complement tradition amplicon-based methods of community profiling. Finally, new techniques such as single cell genomics and metagenome assembled genomes will also help to expand our understanding of polar and alpine cyanobacteria that cannot readily be cultured.

## Teaser

This review discusses how the use of genomics has the potential to greatly expand our understanding of cyanobacteria in cold environments.

## INTRODUCTION

The application of genomic technologies has emerged as a powerful tool in helping to understand the diversity, function, adaptation and evolution of microbes and microbial communities in diverse global environments. In habitats where light and liquid water are readily available, cyanobacteria can make up an important component of these communities, contributing to both carbon and nitrogen fixation and often acting as ecosystem engineers (see Whitton 2012 and chapters therein). Cyanobacteria have had billions of years of evolution (Schirrmeister, Sánchez-Baracaldo and Wacey 2016) and have persisted through profound global environmental including extreme climatic fluctuations and global-scale glaciation (e.g. Neoproterozoic Snowball Earth) (Fairchild and Kennedy 2007). As such, they are highly resilient organisms and have evolved strategies to survive in many extreme environments, with evolutionary radiations into the cryosphere occurring multiple times (Chrismas, Anesio and Sánchez-Baracaldo 2015). In the polar regions, the biomass of vascular plants is reduced with increasing latitude (Walker *et al*. 2016). As such, the relative importance of other photoautotrophs such as cyanobacteria is enhanced, and on glaciers and ice sheets cyanobacteria are responsible for considerable carbon sequestration and driving of the microbial food chain (Anesio *et al*. 2009; Stibal, Šabacká and Žárský 2012). With the advent of -omic technologies (e.g. genomics, metagenomics, transcriptomics and proteomics), the limits of our appreciation of these cyanobacteria mediated processes have expanded from observations and measurements of nutrient fluxes to a much deeper understanding of the molecular mechanisms that allow these processes to take place, while simultaneously shedding light on how cyanobacteria have evolved and adapted to a variety of different ecosystems. For example, the use of genomics (in particular where studies have involved the sequencing of complete or near complete genomes) has expanded our knowledge of cyanobacteria in marine ecosystems by allowing considerable insight into niche differentiation, functional adaptation and biogeography in globally distributed lineages such as *Trichodesmium erythraeum* (Walworth *et al*. 2015), *Crocosphaera watsonii* (Shi *et al*. 2010; Bench *et al*.^2011^), *Synechococcus* spp. (Palenik *et al*. 2003; Palenik *et al*. 2006; Six *et al*. 2007; Scanlan *et al*. 2009) and *Prochlorococcus* spp. (Dufresne *et al*. 2003; Scanlan *et al*. 2009; Coleman and Chisholm 2010; Biller *et al*. 2014; Kashtan *et al*. 2014; Sun and Blanchard 2014; Kent *et al*. 2016). Similarly, whole genome sequences of cyanobacteria from hot springs have helped to elucidate the mechanisms by which they survive in such extreme environments (Bhaya *et al*. 2007; Klatt *et al*. 2011). Genomics studies based on organisms kept in culture collections such as the Pasteur Culture Collection of Cyanobacteria have yielded information about the production of cyanobacterial secondary metabolites (Pancrace *et al*. 2017) and allowed for broad reaching studies covering the entire cyanobacterial phylum (Shih *et al*. 2013). Yet cyanobacteria in the cryosphere have received much less attention at a genomic level, despite having high levels of local ecological importance (Anesio *et al*. 2009; Anesio and Laybourn-Parry 2012).

Until recently, microbial genomics in the cryosphere has been limited to a few studies using metagenomics as a means of evaluating overall community composition or bioprospecting for cold active genes (e.g. Arctic (Choudhari *et al*. 2013), Antarctic (Berlemont *et al*. 2013; Lopatina *et al*. 2016), alpine (Edwards *et al*. 2013)). Much more widespread use has been made of amplicon sequencing. Cyanobacteria specific studies have mainly revolved around the use of SSU rRNA, ITS and LSU rRNA sequences to examine the extent of cyanobacterial diversity in a variety of polar environments including Antarctica (Taton *et al*. 2003; Wood *et al*. 2008; Namsaraev *et al*. 2010) and Svalbard (Strunecký, Komárek and Elster 2012; Pushkareva *et al*. 2015; Palinska, Schneider and Surosz 2017) as well as possible biogeographic links between them (Casamatta *et al*. 2005; Jungblut, Lovejoy and Vincent 2010; Strunecký, Elster and Komárek 2010; Chrismas, Anesio and Sánchez-Baracaldo 2015; Segawa *et al*. 2017). Although these studies are both useful and of considerable interest, they do not address the full extent of functional diversity that only full genome sequences can reveal.

**Figure 1.**
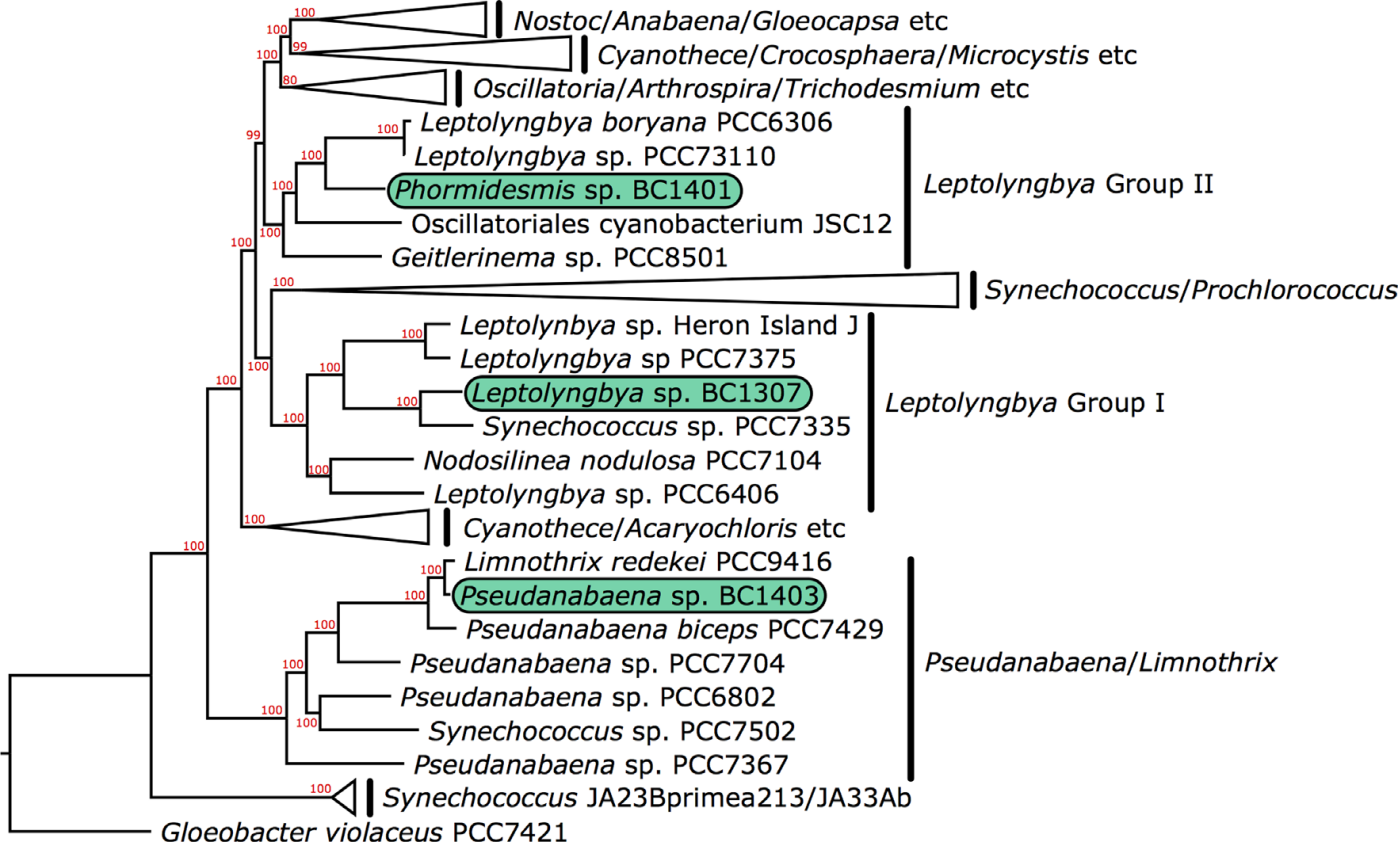
Phylogenomic tree using 136 proteins genes (Blank and Sánchez-Baracaldo 2010) of 95 cyanobacterial taxa indicating the positions of the polar strains *Phormidesmis priestleyi* BC1401, *Pseudanabaena* sp. BC1403 (Greenland, Arctic) and *Leptolyngbya* sp. BC1307 (McMurdo Dry Valleys, Antarctica).

Only now are we truly beginning to look at cyanobacteria from the cryosphere from a genomic perspective (Chrismas *et al*. 2016; Chrismas 2017). *Phormidesmis priestleyi* BC1401 (Accession number: LXYR01000000), *Leptolyngbya* sp. BC1307 (Accession number: NRTA01000000) and *Pseudanabaena* sp. BC1403 (Accession number: PDDM01000000) (Fig. 1) are among the first cyanobacteria from polar environments to have their genomes sequenced and are yielding new information about how cyanobacteria might be adapted to these environments. No genomic indications of true psychrophily were found in these genomes, but genes for other important adaptations such as EPS production, which is implicated in freezing tolerance (Chrismas *et al*. 2016), and mechanisms for tolerating light conditions in Antarctica (Chrismas 2017) were revealed. This work represents the first steps in this area. There are many ways in which the genomics of polar and alpine cyanobacteria might move forward our understanding in a variety of currently underexplored areas including evolutionary biology, functional adaptation to cold environments, regulation and activation of cold associated genes, interactions with viruses and microbial community ecology.

With each year setting a new low point in global glacier coverage (Zemp *et al*. 2015), it is imperative that we explain how key organisms in these environments, such as cyanobacteria, might respond and evolve with anthropogenic climate change. This article serves to outline the prospects for future research into the genomics of cyanobacteria in the cryosphere. We introduce a variety of ways in which genomics can be used to answer biological questions and give examples of how these applications have been previously used. Most of these examples are taken from studies that have used genomics to investigate cyanobacteria in marine ecosystems, although some from more diverse environments such as hot springs are also given. We discuss how these methods can be used to answer specific questions about cyanobacteria in polar and alpine environments.

## GENOMICS OF POLAR AND ALPINE CYANOBACTERIA

### Evolution and adaptation

Recently, phylogenomics (phylogenetic analysis using multiple conserved genes) has emerged as an important tool for understanding the evolution of diverse groups of cyanobacteria over broad timescales and may help us to understand the mechanisms by which cyanobacteria radiated into cold environments. Expanding phylogenies to include more than just the rRNA genes has been shown to give much improved resolution of clades of cyanobacteria such as the *Synechococcus*/*Prochlorococcus* group (Cabello-Yeves *et al*. 2017), unicellular marine diazotrophs such as *Cyanothece, Crocosphaera*, and UCYNA (Bombar *et al*. 2014; Cornejo-Castillo *et al*. 2016) and the Nostocales (Dagan *et al*. 2013; Warshan *et al*. 2017). Furthermore, phylogenomic analyses have allowed for links between cyanobacterial diversification and global scale changes in the environment to be inferred (Larsson, Nylander and Bergman 2011; Schirrmeister *et al*. 2011; Schirrmeister *et al*. 2013; Shih *et al*. 2013; Sánchez-Baracaldo, Ridgwell and Raven 2014; Sánchez-Baracaldo 2015; Schirrmeister, Sánchez-Baracaldo and Wacey 2016). The current lack of genomes from cold environments has prevented true phylogenomic studies of polar and alpine cyanobacteria from being carried out. However, the use of a phylogenomic tree using cyanobacterial genomes from many environments to constrain phylogenies constructed from SSU rRNA sequences of polar and alpine lineages has helped to begin to explain the complexities of cyanobacterial diversity in the cryosphere (Chrismas Anesio and Sánchez-Baracaldo 2015). By using this approach, Chrismas, Anesio and Sánchez-Baracaldo (2015) suggested a mixture of mechanisms for different lineages, varying between (i) ancient cold tolerant ancestors to entire groups of cyanobacteria, and (ii) recent radiations of temperate strains into cryo-ecosystems. Once more genomes from polar and alpine cyanobacteria are available, robust phylogenomic trees can be used to perform more in-depth evolutionary studies and molecular clock analyses. These will help to determine the time that such radiations occurred, and inferring the ecological conditions that prevailed at the time will help us explain how different cold tolerant cyanobacteria originated, while highlighting any links that may exist between the appearance of cold tolerant cyanobacteria and global environmental change.

Arguably one of the most important reasons for generating complete genome sequences of cyanobacteria from cold environments is to better understand how those cyanobacteria are adapted to the variety of environmental pressures of the cryosphere (e.g. freezing, desiccation, high light in summer and low light in winter) (Laybourn-Parry *et al*. 2012), and the implications that such adaptations have for overall ecosystem function (e.g. ecosystem engineering) (Cook, Edwards and Hubbard 2015). While it is becoming increasingly clear from both growth experiments and genomic analysis that cyanobacteria in the cryosphere are not true psychrophiles (Tang and Vincent 1999; Chrismas *et al*. 2016; Chrismas 2017), there is still speculation as to what other mechanisms exist to protect cyanobacteria from the harsh polar and alpine environments, and how those mechanisms first evolved.

Cyanobacteria genomes can be divided into two parts, a ‘stable core’ and a ‘variable shell’ (Shi and Falkowski 2008). The stable core consists of conserved genes needed for essential cellular components such as ribosomes and parts of the photosynthetic apparatus. The variable shell includes metabolically non-essential genes that may confer important environmental adaptations, and may be subject to evolution and periodic loss/acquisition of genes via horizontal gene transfer (HGT) (Mulkidjanian *et al*. 2006; Shi and Falkowski 2008). This flexible shell (also referred to as the pan-genome (Vernikos *et al*. 2015)) also includes strain-specific genes and it is here that we might expect to find the genes responsible for allowing the survival of cyanobacteria in a variety of polar and alpine environments.

Variation of genome content within the cyanobacterial pan-genome that includes ecologically relevant genes has already been demonstrated in marine cyanobacteria. For example, changes in levels of nutrient availability have led to quantifiable differences in the genomes of the picocyanobacteria *Prochlorococcus*, where different ecotypes can be found crossing environmental gradients (Johnson *et al*. 2006; Kashtan *et al*. 2014; Kashtan *et al*. 2017). Differences in phosphorous acquisition genes in *Prochlorococcus* can be seen between the phosphorus rich Pacific and phosphorous deplete North Atlantic (Coleman and Chisholm 2010). A further example of environmentally driven changes in physiological capabilities can be seen in *Synechococcus* isolated from alkaline siliceous hot springs, which contained ferrous iron transport related genes not present in a related reference genome (Klatt *et al*. 2011). Conversely, considerable variation in genomic amino acid identity can also occur between closely related organisms with the same functional and ecological role such as in the symbiotic diazotroph UCYN-A (Bombar *et al*. 2014). Changes in gene complement such as the examples above highlight the plasticity of cyanobacterial genomes, and how knowledge of genomic variation within both lineages and populations is fundamental to our understanding of how cyanobacteria interact with, and contribute to, the environment.

Exactly what these adaptations are in polar and alpine cyanobacteria requires further investigation. The production of EPS is known to confer freezing and desiccation tolerance in cyanobacteria (Tamaru *et al*. 2005; Knowles and Castenholz 2008) and the genes for EPS production have already been identified in *Phormidesmis priestleyi* BC1401 (Chrismas *et al*. 2016), but how they are regulated or vary between cold tolerant lineages across the cyanobacterial phylum is as yet unknown. Ice binding proteins (IBPs) are another key adaptation in ice dwelling organisms. IBPs are a diverse group of proteins (Davies 2014; Bar Dolev, Braslavsky and Davies 2016) that prevent ice nucleation and have been found in bacteria from polar environments such as sea ice (Raymond, Fritsen and Shen 2007) and cryoconite (Singh *et al*. 2014). Antarctic cyanobacterial mats have also been shown to inhibit ice crystal formation, a property not seen in temperate cyanobacterial mats (Raymond and Fritsen 2001), and combined proteomic and genomic approaches will help to understand the importance of IPBs in cold adapted cyanobacteria. Fatty acid desaturation represents another mechanism of cold tolerance in cyanobacteria, helping to maintain membrane fluidity at low temperatures (Murata and Wada 1995; Chintalapati, Kiran and Shivaji 2004). Fatty acid desaturase gene complement varies across the cyanobacterial phylum (Chi *et al*. 2008) and, by expanding upon the number of available genomes of polar cyanobacteria, it will be possible to see the full extent that desaturase genes vary in cold tolerant lineages. Other adaptations unrelated to cold but needed for withstanding other environmental stressors such as light are also important. Antarctic eukaryotic algae are adapted to survival under polar light regimes (Morgan-Kiss *et al*. 2006; Morgan-Kiss *et al*. 2015), and there is evidence for adaptation of the light harvesting complex in the Antarctic *Leptolyngbya* sp. BC1307 (Chrismas 2017); combining genome interrogation with photophysiology experiments will therefore be key to explaining light adaptation in these organisms. There are doubtless other adaptations yet to be discovered, and investigating the presence of these ecologically important genes in polar and alpine cyanobacteria is essential to understanding how they have evolved to survive in such extreme environments.

### Biogeography and population genomics

**Figure 2.**
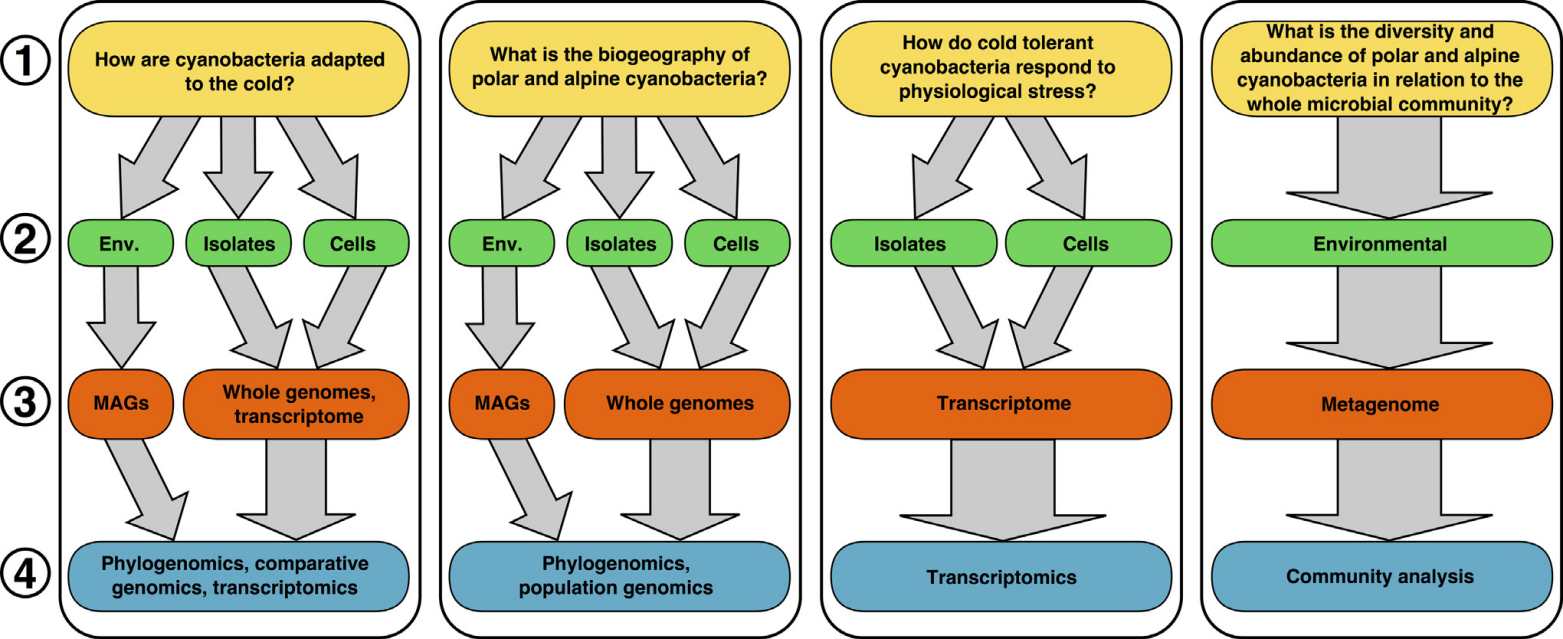
Potential methods to be used for investigating (1) important questions regarding polar and alpine cyanobacteria, including (2) sampling type (e.g. environmental samples, isolated strains or single cells/filaments), (3) data generated (e.g. metagenomes, metagenome assembled genomes (MAGs), whole genomes or transcriptomes) and (4) analytical methods required (e.g. analysis of community composition, phylogenomics, comparative genomics or transcriptomics).

There is considerable scope to investigate genomic differentiation within lineages that are only found in cold habitats that can tell us about both adaptation and biogeography of these organisms. For example, *Phormidesmis priestleyi* is an ecologically important cyanobacterium that can be found both in the Arctic and Antarctica (Komárek *et al*. 2009; Chrismas, Anesio and Sánchez-Baracaldo 2015) with highly similar SSU rRNA sequences between populations from either side of the globe. Investigating genomic variability between organisms isolated from the Arctic and Antarctica such as *Phormidesmis priestleyi* BC1401 (Chrismas *et al*. 2016) and *Phormidesmis priestleyi* ULC007 (Lara *et al*. 2017) may tell us a great deal about similarity between these geographically distant but ecologically related environments (Fig. 2). Additionally, many lineages of cyanobacteria found in the cryosphere (e.g. *Nostoc*, *Leptolyngbya, Chroococcidiopsis*) are thought to be cosmopolitan with some members of a lineage being found in cold habitats, while others may exist in temperate, tropical or arid environments (Bahl *et al*. 2011; Chrismas, Anesio and Sánchez-Baracaldo 2015). Comparing between the genomes of organisms from these distinct populations can yield information into subtle adaptive changes between them depending on the prevailing ecological conditions. For example, variation in the photosynthetic genes in *Leptolyngbya* sp. BC1307 compared to closely related lineages suggests the ability to account for light conditions in Antarctic terrestrial environments (Chrismas 2017). Many other such adaptations are likely to exist and by expanding the number of sequenced genomes of cyanobacteria from the cryosphere and further identifying genomic components likely to be under selection in cold environments,we may begin to observe ecological differentiation within lineages found both in and out of cold environments that is masked by closely related SSU rRNA sequences.

Environmental gradients within an ecosystem can also be big drivers for both changes in cyanobacterial community structure (Bolhuis, Fillinger and Stal 2013) and genomic diversification (Koza *et al*. 2011; Hahn *et al*. 2016). Many types of environmental gradient can be seen in the cryosphere, and they have been shown to influence microbial community composition and drive interspecific, or between species, variation in cyanobacteria. For example, on the Tibetan Plateau over an elevation gradient of 5300 m–5900 m, the relative abundance of cyanobacteria has been shown to shift in response to an increase in elevation and a decrease in phosphorous and nitrogen availability (Janatková *et al*. 2013). Similar changes in microbial community structure caused by local environmental conditions such as nutrient availability (Logares *et al*. 2013; Borghini *et al*. 2016) and oxygen gradients (Jungblut *et al*. 2016) have been demonstrated in Antarctic lakes, in the Arctic along developing soils in pro-glacial moraines (Hodkinson, Coulson and Webb 2003; Kwon *et al*. 2015) and in the Alps in recently isolated proglacial lakes (Peter and Sommaruga 2016). While interspecific differences in polar cyanobacterial communities in these situations are well documented, much less is understood about intraspecific or within species variation. In many of the situations described above, certain key cyanobacteria (e.g. *Leptolyngbya* and *Phormidesmis*) can be found in varying abundance across the entire environmental gradients, yet the extent to which these organisms vary at a genomic level is unknown.

Population genomics, which deals with genome wide variation within lineages to understand biogeographical links and the extent of dispersal between populations, will be of great importance in understanding how distinct populations of polar and alpine cyanobacteria interconnect. Evidence for population structuring within the SSU rRNA and ITS sequences of several lineages of cryoconite cyanobacteria has already been shown (Segawa *et al*. 2017), and by expanding this to look at differences across the genome, a much clearer resolution of cyanobacterial biogeography will become apparent. Single cell sequencing is now emerging as the optimal way of doing population genomics (Kashtan *et al*. 2014) as it has the benefit of producing an entire genome and single nucleotide polymorphisms (SNPs) for a single cell. Methods of cell isolation are more complex than culture-based techniques, with cells being captured by a variety of methods including flow cytometry, microfluidics and dilution to extinction. However, alternative approaches to cell isolation may be applicable to cyanobacteria. Hayes *et al*. (2002) successfully carried out PCR of specific loci on single filaments of *Nodularia* derived from natural populations in the Baltic sea allowing for SNP analysis in single genes. This might be a suitable approach for cyanobacteria from the cryosphere since many cold environments are dominated by filamentous lineages; when combined with whole genome amplification and sequencing this could represent an efficient way of investigating population level diversity in filamentous lineages of polar and alpine cyanobacteria without the need to resort to cell isolation methods requiring expensive specialized equipment. Alternatively, new bioinformatics approaches are improving on our ability to generate metagenome assembled genomes (MAGs). This allows draft genomes to be obtained directly from environmental samples (Hugerth *et al*. 2015; Parks *et al*. 2017; Tully *et al*. 2017) and has the potential to greatly increase the number of available cyanobacterial genomes directly linked with specific geographical locations. Together, these methods will make it possible to use cyanobacteria in the cryosphere as model systems for investigating mechanisms of microbial evolution, including environmental adaptation, rates of genomic evolution and the extent of gene flow and recombination within and between populations.

### Response to physiological stress

As discussed in the previous section, evidence is growing to support the fact that cyanobacteria from the cryosphere are not true psychrophiles. The overwhelming majority of cultured cold cyanobacteria have thermal optima well above their ambient environments (Tang, Tremblay and Vincent 1997), and there are no clear genomic signatures of cold adaptation in the genomes of the Arctic *Phormidesmis priestleyi* BC1401 and the Antarctic *Leptolyngbya* sp. BC1307 (Chrismas *et al*. 2016; Chrismas 2017). Yet these and other cyanobacteria are still capable of withstanding the intense environmental pressures of the polar regions. The mechanisms for doing so may, therefore, lie in the regulation of existing mechanisms that are common throughout the group (Chrismas *et al*. 2016; Sinetova and Los 2016a).

Levels of gene expression are known to vary dramatically under different environmental conditions with upregulation of stress response genes being a prime example of this. In cyanobacteria, iron limitation (Ludwig and Bryant 2012; Kopf *et al*. 2014), light stress (Billis *et al*. 2014; Kopf *et al*. 2014; Kopf *et al*. 2015), salt (Billis *et al*. 2014; Al-Hosani *et al*. 2015), and nitrogen limitation (Kopf *et al*. 2014; Choi *et al*. 2016) among others all initiate expression of groups of specific genes in order to alleviate the cellular stress that these environmental pressures incur. Cold stress is no different. In microarray expression experiments, over 100 genes in *Synechocystis* were upregulated by a factor of at least two under cold stress (although only 38 of these being exclusively implicated in cold stress response) (Sinetova and Los 2016b). Since most cyanobacteria from the cryosphere do not grow preferentially at low temperatures (Tang, Tremblay and Vincent 1997) (except for Ant-Orange (Nadeau and Castenholz 2000)), they are likely to be experiencing constant stress in their environment. However, constant expression of cold shock genes is likely to be metabolically expensive and as such cells may be acclimated to growth at low temperatures rather than exhibiting a persistent cold shock response. Determining the time and rate at which cold response genes in cold-adapted cyanobacteria are expressed is therefore essential if we are to understand how these organisms survive. Where the mechanisms allowing cold tolerance are common throughout the cyanobacterial phylum (e.g. the production of EPS, Pereira *et al*. 2015; fatty acid desaturase genes, Chi *et al*. 2008), differences may exist in the way these shared characteristics are regulated to account for the increased levels of expression required in cold environments. Indeed, in Antarctic *Nostoc* sp., constitutive expression of desaturase genes has been observed rather than being upregulated upon temperature reduction, as is seen in temperate lineages (Chintalapati *et al*. 2007). Identifying how these processes are regulated is therefore key to explaining mechanisms of long term cold tolerance.

Investigation into the cyanobacterial transcriptome can take one of two forms. Either a global transcriptome can be generated under stress conditions (e.g. Ludwig and Bryant 2012; Harke and Gobler 2013; Teikari *et al*. 2015) to show all genes transcribed at any given time, or RNA-sequencing can be targeted at key transcripts or regulatory RNAs of interest. The availability of the genomes of key organisms is essential for this; by identifying key genes of interest within the genome (e.g. the genes responsible for the production of EPS in *Phormidesmis priestleyi* BC1401, Chrismas *et al*. 2016, or genes involved in cold shock response), transcriptomic studies can be targeted towards these genes and their putative regulatory networks to establish how cold tolerant cyanobacteria might be reacting to their environment at a molecular level.

### Community ecology and interactions

While cyanobacteria often dominate the habitats that they inhabit in the cryosphere, they do not exist in isolation within their environment. Instead, they are members of complex communities containing multiple types of cyanobacteria, heterotrophic bacteria and eukaryotes (e.g. Gordon *et al*. 2000; Paerl, Pinckney and Steppe 2000; Torre *et al*. 2003; Jungblut *et al*. 2012; Edwards *et al*. 2013; Ambrosini *et al*. 2017). Determining the structure of these communities is fundamental to understanding the overall function of microbially dominated cryo-environments. Typically, SSU rRNA gene amplicon-based approaches have been used to determine abundances of different cyanobacteria within cryospheric environments. Such techniques have been used to show that the relative abundance of the cyanobacterial component of snow communities varies from site to site in alpine snow (Wunderlin, Ferrari and Power 2016), and revealed regional scale variation in cryoconite communities from glaciers on the Tibetan Plateau (Liu *et al*. 2017). Community profiling such as this has revealed many insights into how cyanobacteria interact with the environment. These include how cyanobacterial abundance varies in response to soil development and abiotic factors like pH (Pushkareva *et al*. 2015), succession in glacial forefields (Knelman *et al*. 2012; Rime *et al*. 2015), and changes in altitude and nutrient composition as discussed earlier (Janatková *et al*. 2013; Logares *et al*. 2013; Borghini *et al*. 2016; Jungblut *et al*. 2016). However, PCR bias is known to influence the resolution and sensitivity of operational taxonomic unit (OTU) recovery, and metagenomics can recover 1.5 to ∼10 × more phyla than amplicon-based approaches (Poretsky *et al*. 2014). The extent of cyanobacterial diversity (indeed, all microbial diversity) in the cryosphere is therefore likely to be much greater than is presently known, which has considerable implications for how we interpret microbial ecology in the cryosphere and associated biogeochemical processes. Expanding on existing studies with metagenomics is therefore essential if we are to know the true diversity of both polar cyanobacteria and their associated microbial communities, and the ability to obtain cyanobacterial MAGs from these metagenomes will allow for deeper investigation of cyanobacteria that cannot be isolated or cultured using traditional methods (Hugerth *et al*. 2015; Parks *et al*. 2017; Tully *et al*. 2017).

Understanding how members of these communities interact with each other is also of great importance. For example, laminated cyanobacterial mats (such as those common in polar and alpine environments) include layers of methanogens and sulfur-reducing bacteria (Stal 1995; Bolhuis, Fillinger and Stal 2013) that interact to form a network of metabolic interdependencies. In many cases, such interactions are essential for survival, and when cyanobacteria are removed from their community, growth can sometimes be impaired or inhibited altogether (Xie *et al*. 2016). Multi-omic techniques can help us understand these community interactions (Franzosa *et al*. 2015). Differences in transcriptional regulation were observed in different strains of *Prochlorococcus* when they were grown in co-culture with marine *Alteromonas* (Aharonovich and Sher 2016), and metagenomics has revealed that *Microcystis* is dependent on associated microbiota for Vitamin B12 synthesis (Xie *et al*. 2016). The extent to which cyanobacteria are reliant upon the community and vice versa is a therefore a key question in microbial ecology, and cyanobacteria-dominated communities in the cryosphere represent excellent systems for investigating these kinds of community interactions. In particular, cryoconite holes act as a semi-closed system of cyanobacteria-dominated microbial communities that may be investigated in the field or reproduced in the lab.

Another important ecological interaction in polar environments is the cyanobacteria-fungus symbiosis in cyanolichens. Cyanobacteria are the main photobiont in several Arctic lichens such as *Peltigera*, *Solorina* and *Nephroma* spp. (Rikkinen 2015) and *Peltigaria* spp. have also been shown to exhibit considerable diversity in maritime Antarctica (Zúñiga *et al*. 2015). Lichen cyanobionts are primarily diazotrophs such as *Nostoc* and *Stigonema*, which can also exist as nitrogen fixing symbionts alongside green algal photobionts in tripartite lichens (Rozema, Aerts and Cornelissen 2007). The importance of cyanolichens for nitrogen fixation in Arctic environments is clear; Weiss, Hobbie and Gettel (2005) showed that abundance of *Peltigaria aphthosa* in tundra was diminished when an eternal source of nitrogen was added, and cyanolichen-mediated nitrogen cycling is likely to have widespread ecosystem implications in the Arctic (Wookey *et al*. 2009). However, the molecular biology of the cyanobacteria-fungus symbiosis is still developing (Rikkinen 2013) and genomic approaches have great potential to improve our understanding of polar cyanolichens in terms of their evolution and ecology. Metagenomics can be used to investigate the entire lichen consortium (Grube *et al*. 2013), which can shed light on the genomic composition of not only the main symbiotes but also the associated microbiome (Bates *et al*. 2011; Sigurbjörnsdóttir, Andrésson and Vilhelmsson 2015). Investigating the cyanobiont alone may also provide interesting evolutionary insights. Genome reduction is common in symbiotic prokaryotes (McCutcheon and Moran 2012) including cyanobacteria (Bombar *et al*. 2014), and by sequencing cyanobiont isolates from cyanolichens we may better understand the extent of the symbiotic relationship. However, culturing the cyanobiont from lichens is not trivial. Isolates obtained from cyanolichens are often found not to be the main photobiont (Summerfield, Galloway and Eaton-Rye 2002), and using new single cell genomics approaches may be of help here.

It is well understood that interactions with viruses are an important part of the processes that drive the evolution of microbial genomes (Weinbauer and Rassoulzadegan 2004), and the same is likely to hold true for cyanobacteria in the cryosphere. It has been proposed that viruses are one of many factors that contribute to the evolution of cyanobacteria (Shestakov and Karbysheva 2015) and direct interactions of viruses with cyanobacterial genomes are fundamental to this. Genes native to cyanobacteria (e.g. genes involved in the cyanobacterial photosynthetic apparatus) are regularly found within the genomes of cyanophage (Sullivan *et al*. 2005) and the genomes of some cyanobacterial T4-like myophage have been found to be significantly shaped by their host organism (Ignacio-Espinoza and Sullivan 2012). Likewise, cyanophage help to mold the genomes of their cyanobacterial hosts (Coleman *et al*. 2006; Lindell *et al*. 2007) and virus-mediated HGT is thought to be responsible for the acquisition of novel genes and may be involved in the rearrangement of genome structures (Kuno, Sako and Yoshida 2014). There is considerable potential for these processes to be acting on cyanobacteria from the cryosphere (Anesio and Bellas 2011; Rassner *et al*. 2016). Viruses are found at relatively high numbers in cryoconite holes. In cryoconite from Greenland and Svalbard, abundance of viruses was between 5.62 × 10^8^ (Midtre Lovénbreen, Svalbard) and 24.5 × 10^8^ (Greenland Ice Sheet, 11 km) virus-like particles per gram of dry weight sediment (Bellas *et al*. 2013). The assembly of potential virus genomes from the virus size fraction of these cryoconites resulted in four scaffolds of viruses that had putative cyanobacterial hosts (Bellas, Anesio and Barker 2015) and the genomes of eight viruses were recovered from cyanobacterial mats on the McMurdo Ice Shelf (Zawar-Reza *et al*. 2014). A high proportion of cyanobacterial cells in various Antarctic lakes were observed to contain prophage (Säwström *et al*. 2007). In some cases, these viruses may be specific to polar cyanobacteria and a novel lineage of cyanophage, S-EIV1, was found to infect Arctic *Synechococcus* (Chénard *et al*. 2015).

Modern genomics techniques can be used to investigate the influence of these abundant viruses on cyanobacteria in the cryosphere. Metagenomes can be used to link virus-host interactions in cyanobacterial mats (Voorhies *et al*. 2016) and once a cyanobacterial genome has been sequenced, detecting past virus-host interactions is possible due to viruses leaving distinct signatures on microbial genomes. Insertion elements can be evidence of HGT, while clustered regularly interspaced short palindromic repeats (CRISPRs) are evidence of previous exposure to viruses. More direct evidence might be found in the form of *in situ* prophage integrated into a cyanobacterial genome (Chénard, Wirth and Suttle 2016). With the availability of new genomes of cyanobacteria from the cryosphere, our knowledge of the effect of viruses in cyanobacterial dominated ecosystems will increase correspondingly.

## CONCLUSION

Genomics is no longer next-generation science; it is both contemporary and essential. The advances that large-scale genomics projects have had on our understanding of marine cyanobacteria have been substantial, leading to new discoveries in terms of their global biodiversity and biogeochemistry. The earth's polar and alpine regions are ripe for expanding these techniques into extreme environments that have been hitherto underexplored in respect to cyanobacterial genomic diversity. By sequencing many genomes of single cyanobacterial lineages from diverse polar and alpine habitats, we may better understand their population structure and begin to answer questions about biogeography, dispersal and functional adaptation. Such genomes will help to complement metagenomic and transcriptomic approaches allowing us to better understand their role in polar microbial communities and how they might react to environmental pressures. In a changing climate where the extent of glaciers is in widespread decline, efforts should be made how these organisms might respond to these changes from both an evolutionary and ecological perspective.
